# Highly Porous Fluorapatite/β-1,3-Glucan Composite for Bone Tissue Regeneration: Characterization and In-Vitro Assessment of Biomedical Potential

**DOI:** 10.3390/ijms221910414

**Published:** 2021-09-27

**Authors:** Leszek Borkowski, Agata Przekora, Anna Belcarz, Krzysztof Palka, Mariusz Jojczuk, Piotr Lukasiewicz, Adam Nogalski, Grazyna Ginalska

**Affiliations:** 1 Chair and Department of Biochemistry and Biotechnology, Medical University of Lublin, Chodzki 1, 20-093 Lublin, Poland; anna.belcarz@umlub.pl (A.B.); g.ginalska@umlub.pl (G.G.); 2 Independent Unit of Tissue Engineering and Regenerative Medicine, Chair of Biomedical Sciences, Medical University of Lublin, Chodzki 1, 20-093 Lublin, Poland; agataprzekorakusmierz@umlub.pl; 3 Faculty of Mechanical Engineering, Lublin University of Technology, Nadbystrzycka 36, 20-618 Lublin, Poland; k.palka@pollub.pl; 4 Chair and Department of Traumatology and Emergency Medicine, Medical University of Lublin, Staszica 11, 20-081 Lublin, Poland; mariuszjojczuk@tlen.pl (M.J.); piotr.lukasiewicz1@umlub.pl (P.L.); noad@tlen.pl (A.N.)

**Keywords:** cell growth evaluation, composite material, cytotoxicity, fluorapatite, osteogenic differentiation

## Abstract

A novel fluorapatite/glucan composite (“FAP/glucan”) was developed for the treatment of bone defects. Due to the presence of polysaccharide polymer (β-1,3-glucan), the composite is highly flexible and thus very convenient for surgery. Its physicochemical and microstructural properties were evaluated using scanning electron microscopy (SEM), Fourier transform infrared spectroscopy (FTIR), mercury intrusion, mechanical testing and compared with the reference material, which was a hydroxyapatite/glucan composite (“HAP/glucan”) with hydroxyapatite granules (HAP) instead of FAP. It was found that FAP/glucan has a higher density and lower porosity than the reference material. The correlation between the Young’s modulus and the compressive strength between the materials is different in a dry and wet state. Bioactivity assessment showed a lower ability to form apatite and lower uptake of apatite-forming ions from the simulated body fluid by FAP/glucan material in comparison to the reference material. Moreover, FAP/glucan was determined to be of optimal fluoride release capacity for osteoblasts growth requirements. The results of cell culture experiments showed that fluoride-containing biomaterial was non-toxic, enhanced the synthesis of osteocalcin and stimulated the adhesion of osteogenic cells.

## 1. Introduction

Limitations related to native or exogenous bone transplants acquirement and accompanying complications (e.g., graft necrosis as a consequence of lack of blood supply, formation of a defect in a new site, adverse immune reactions resulting in incomplete healing of grafts, the possibility of transmitting an infectious agent) increase the importance of bone substitutes, i.e., bone-substituting biomaterials, in orthopaedic procedures. Bioceramics based on calcium phosphate (CaP) are widely used in the field of bone regeneration, both in orthopaedics and dentistry, due to their good biocompatibility, bioactivity, osseointegration and osteoconduction [1]. Bone substitutes such as hydroxyapatite (HAP), tricalcium phosphates (α- and β-TCP) or bioactive glasses (BG) are therefore an alternative to auto- or allogeneic bone grafts [2,3]. These CaP materials, due to their characteristics, promote the migration of bone cells to their interior and the vascularization of the implant, which facilitates the process of bone tissue reconstruction [4,5]. However, ceramic materials exhibit the disadvantages such as low fracture toughness and poor surgical convenience. The above limitations can be reduced or even eliminated by adding another component, e.g., a synthetic polymer (polylactide, polyglycolide, polycaprolactan) or natural polymer (collagen, gelatin, elastin or polysaccharides: chitosan, glucan), to form a composite material. The composites combine the ductility and flexibility of the polymer phase with the strength and biocompatibility of the ceramic phase and, as a result, the produced composites become more advantageous in medical applications [6,7,8,9,10,11,12,13]. The use of different types of natural fiber biocomposite scaffolds for fractured bone repair were reviewed by Jouyandeh et al., 2021 [14].

Recently, calcium-phosphate-fluoride ceramics (fluorapatite, FAP) has attracted attention in the area of bone tissue regeneration. Recent studies comparing fluorapatite and hydroxyapatite, in relation to their medical applications, have shown that FAP exhibits relatively higher thermal stability and better mechanical properties than HAP [15]. Some biological tests also indicated better biological properties of fluoridated apatites (fluorapatite, fluorhydroxyapatites (FHAPs)) than those of HAP. Tredwin et al., observed an improved cell proliferation (expressed as a lower number of dead cells) on FAP and FHAP surface, compared to HAP surface [16]. In the studies of Wu et al. [17], it was found that fluorapatite can increase collagen synthesis. Moreover, Bhadang et al. [18] showed that FAP integrates well with bone tissue. According to Harrison et al. study [19], the viability of cells in the medium incubated for 48 h with FAP material was higher than in the medium incubated with HAP. Some studies show that F^−^ released from fluoride-containing bone implants can stimulate the proliferation and differentiation of osteoblasts [20,21] and accelerate the bone mineralization process [22]. For these reasons, the use of fluoride-substituted hydroxyapatite in the treatment of damaged bone tissue may be promising.

So far, fluorapatite has been used, among others, as a component of tooth filling cements [23], as an intermediate layer between HAP and ZrO_2_ [24] and as coatings on metallic implants, mainly made of titanium [25,26,27]. For in-vivo experiments, FAP-coated titanium materials were implanted into the jaws of dogs [28] and long bones of goats [29]. These studies confirmed that FAP promoted the formation of bone tissue and also reduced the resorption of the ceramic coating on implants in relation to HAP.

It is noteworthy that currently on the medical market there is no surgically convenient bone substitute material that would also carry fluoride ions, released in specific amounts directly at the bone defect, in order to accelerate the mineralization process and stimulate osteogenic cells. Such material, while being a scaffold for osteoblasts, could act as an intensifier of the bone tissue regeneration process. Therefore, the aim of the present work was to develop a bioresorbable composite bone scaffold based on the polysaccharide polymer and fluorapatite granules produced by our team according to the method described in the Polish Patent no. 235803 [30]. Our previous research showed that the modified sol-gel method allowed for the successful introduction of fluoride ions into the structure of the apatite network, and the obtained fluorapatite, after calcination at 800 °C, was characterized by a gradual release of F^−^ at a level appropriate for the osteoblast cell line and supporting osteoblast proliferation [31]. Therefore, FAP has been used in our present work to prepare a ceramic-polymer FAP/glucan composite material with properties, which would potentially support regeneration of bone tissue. It is worth noting that FAP/glucan scaffold and the method for its production is novel and described in Polish Patent no. 236369 [32]. Fluoride-free HAP/glucan composite was used as a reference material. Physicochemical and microstructural properties of produced materials were evaluated by mercury intrusion porosimetry, SEM, FTIR, and mechanical testing. Then, in-vitro bioactivity, ion reactivity, absorbability, and fluoride release were assessed. Finally, cell culture experiments were carried out, i.e., MTT viability test, fluorescent staining of cells grown on the scaffolds (Live-Dead, Phalloidin/DAPI) and ELISA tests (osteogenic differentiation markers: collagen, osteocalcin and bone alkaline phosphatase).

## 2. Results and Discussion

**Figure 1:**
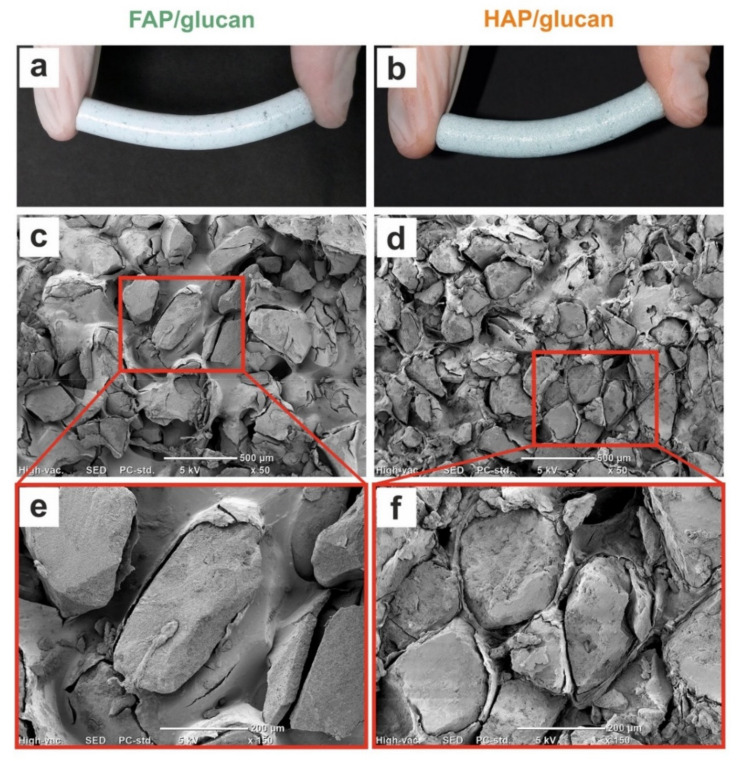
Panels (**a**,**b**) show macroscopic images of FAP/glucan and HAP/glucan after squeezing in fingers. Panels (**c**,**d**) show SEM images of the surface of materials magnified 50×. Panels (**e**,**f**) show SEM images of magnified fragments of composites surface (marked with a red frame on panels **c**,**d**).

In multicomponent materials for bone defects, apatite intensifies the bioactivity and osteoconductivity, while polymer enhances mechanical properties and biocompatibility. It was observed for biomaterials containing collagen, chitosan or alginate as polymeric compound [33,34,35,36]. In our study, both curdlan-containing composites were elastic (they may be bent and squeezed when wet and retain their original shape after the strength removal) in comparable way (Figure 1a,b). These results are in agreement with the observations concerning the elasticity of HAP-ceramics/curdlan composite [37].

SEM images of dry FAP granules-based and HAP-granules-based (reference) composites, containing β-1,3-glucan as a binder, were presented in Figure 1c–f. It was clearly shown that both composites contain irregular in shape ceramic granules embedded in polymeric network of curdlan. In both cases granules of irregular shape were tightly surrounded by the layer of polymerized curdlan which also fills the space between the ceramics (Figure 1c–f). This enables the tight connections between polymeric and ceramic compounds and allows both dry and soaked biomaterials to keep their shape and integrity. Moreover, it seems that distribution of granules and polymer in both biomaterials is different. Some granules in FAP/glucan composites are larger than granules in HAP/glucan composites, similarly the areas covered by polymeric phase (Figure 1e,f).

**Figure 2:**
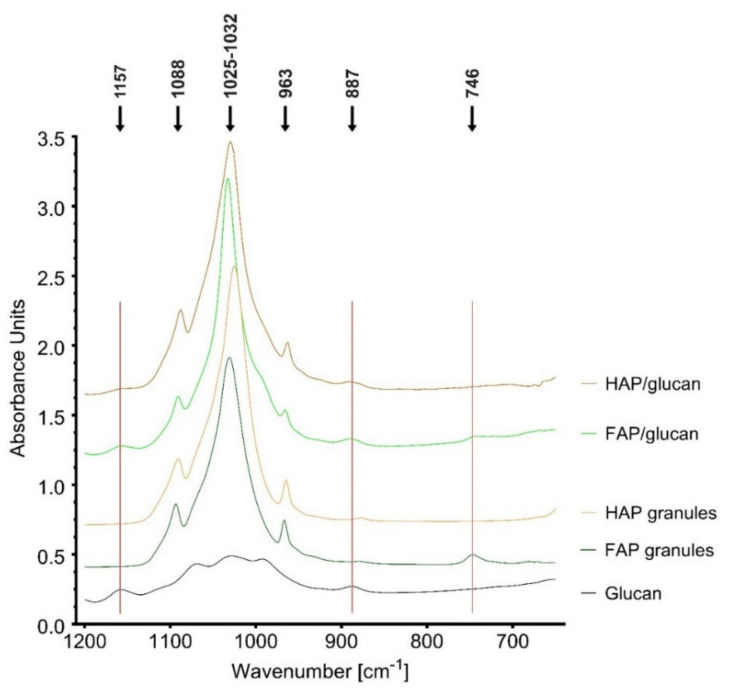
FTIR spectra of the composites, ceramic granules and polysaccharide polymer.

Figure 2 shows the most characteristic part of FTIR spectra of tested materials (1200-650 cm^−1^). Bands at 963 cm^−1^, 1025-1032 cm^−1^ and 1088 cm^−1^, indicating the presence of phosphate groups, are visible in spectra of both composites and granules. Moreover, band at 746 cm^−1^ (indicating the presence of F^−^ ions) in spectrum of FAP granules is present also in the spectrum of FAP/glucan composite [31]. Bands of C_1_-O-C_3_ stretching vibrations at 887 cm^−1^ and 1157 cm^−1^ (characteristic for β-glucan) are found in spectra of both glucan and the composites, confirming the presence of curdlan in created biomaterials. No additional bands, suggesting the appearance of chemical interactions between curdlan and ceramics, appeared in the composite spectra. This suggests that the mechanism of FAP/glucan composite is based only on physical occlusion of granules in polymeric network, as observed previously [37]. 

**Figure 3:**
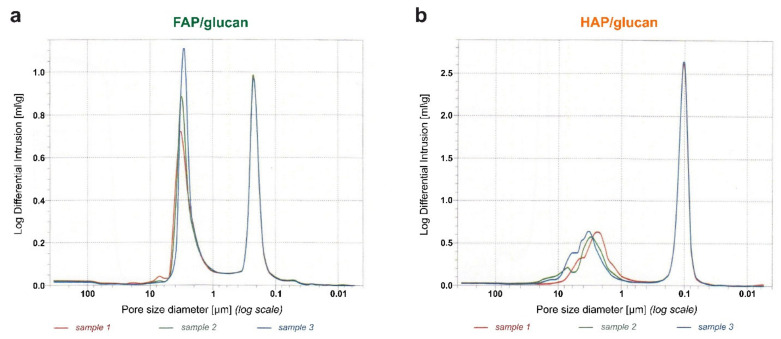
Pore size distribution expressed by the log-differential intrusion volumes (presented on logarithmic scales) for the FAP/glucan (**a**) and HAP/glucan (**b**) composites.

Porosity parameters and pore size distribution of tested composites were shown in Figure 3 and Table 1. Total porosity of the prepared FAP-based and HAP-based composites is high in both cases and varies between 53% for FAP-based one and 66% for HAP-based one (Table 1). Much more significant differences were observed for total pore surface areas of the tested composites: that of FAP/glucan biomaterial is four times lower than that of HAP/glucan one (Table 1). Both these parameters suggest that FAP/glucan composite is more dense and less porous than HAP/glucan composite. This feature is most probably related to the properties of fluorapatite, the main compound of the composite. It was reported that bulk density and porosity of FAP granules are 0.75 g/cm^3^ and 52% in comparison with 0.55 g/cm^3^ and 64% for HAP granules [31]. Volume of intrinsic pores in FAP and HAP granules was 0.36 cm^3^/g and 0.58 cm^3^/g, respectively [31]. Relation between the density and fluoride content in ceramics was also reported by Gross et al. [38]. They observed that density of pellets of pure hydroxyapatite and fluorapatite sintered at 1200–1250 °C were similar. But when HAP and FAP were sintered at lower temperature (1150 °C), fluorapatite pellets were denser than that of HAP [38]. Other studies of the properties of fluorhydroxyapatites (FHA) with different level of fluoride ions indicated an increase in density of samples with an increase in fluoride ions [39,40]. Therefore, the results of our experiments are in agreement with other available reports on porosity of fluorapatite ceramics.

**Figure 4:**
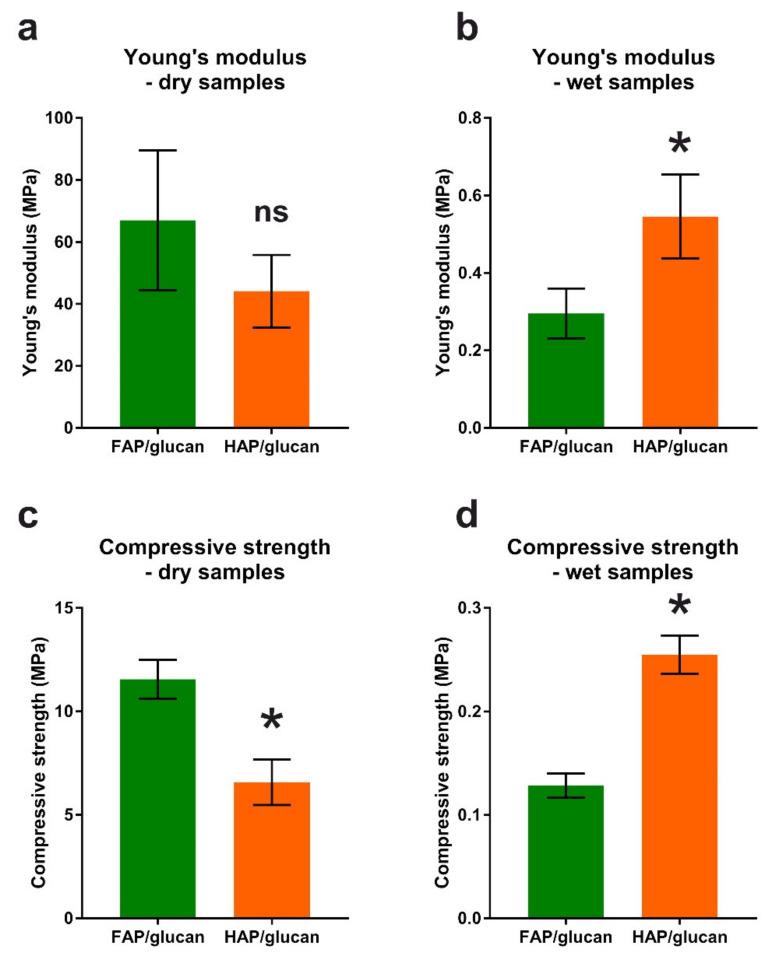
Mechanical parameters of composites measured in a dry state and after a 24-h soaking in deionized water. *, statistically significant difference between FAP/glucan and HAP/glucan composites according to unpaired Student’s *t*-test; ns, no significant.

Mechanical parameters of FAP/glucan and reference HAP/glucan composite were evaluated both for dry and wet samples (soaked in deionized water for 24 h). This was logical because—after implantation in the human body—the porous composites will absorb tissue liquid and will become elastic. Young’s modulus reflects the susceptibility of tested material to deform under mechanical stress. As shown in Figure 4, Young’s modulus for dry FAP/glucan composite is higher (67 MPa) than that for HAP/glucan composite (44 MPa). Therefore, FAP-based composite undergoes deformation less than the HAP-based one. This was expected as FAP granules are denser and may show higher resistance to deformation. It is clear that biomaterial in dry state, composed of ceramic granules in 92%, the ceramics is the main factor responsible for mechanical properties. However, for soaked samples, the relation is opposite: Young’s modulus for FAP/glucan composite is lower (0.3 MPa) than that for HAP/glucan composite (0.55 MPa). Compressive strength values of tested composites show similar tendency. For dry FAP/glucan composite it is higher (11.55 MPa) than that for HAP/glucan composite (6.57 MPa), while for wet materials the opposite relation is observed: 0.13 MPa and 0.25 MPa, respectively (Figure 4).

Both tested mechanical parameters (Young’s modulus and compressive strength) are lower for soaked materials than for dry composites. Most probably the explanation of this phenomenon is the hydration of curdlan phase. Natural polymers, as reported for example for alginate, exhibit significant reduction of mechanical properties after hydration: alginate’s Young’s modulus drops after the hydration from 292 MPs to 49.7 MPa [41]. It should be noted that hydrated polymeric phase becomes dominant in determination of mechanical parameters of ceramic/glucan composites. It may be surprising that mechanical parameters are higher for FAP/glucan biomaterial than for HAP/glucan when they are dry; after soaking this relation is reverse. This phenomenon may be explained by the differences in granules distribution in FAP/glucan and HAP/glucan composites. As shown in Figure 1, the range of granules size in FAP/glucan material is wider than in HAP/glucan composite (some of the granules are larger than in HAP/glucan material). This may create also wider interspaces occupied by polymeric network between the granules and—after swelling in aqueous solutions—may affect mechanical properties of wet composites.

According to Misch et al., the compressive strength of the human mandibular trabecular bone ranged from 0.22 to 10.44 MPa, with a mean value of 3.9 MPa (SD = 2.7) and the Young’s modulus ranged from 3.5 to 125.6 MPa, with a mean value of 56.0 MPa (SD = 29.6) [42]. Based on these data, it can be concluded that the values of the compressive strength and the elastic modulus of both tested biomaterials in the dry state are at a similar level. On the other hand, in the wet state (e.g., after implantation into the body), both biomaterials are more flexible than the spongy bone and less resistant to compression. For this reason, we propose the use of new bioactive and highly porous material for filling bone defects in order to accelerate the regeneration process and not as a permanent bioinert replacement for bone tissue.

**Figure 5:**
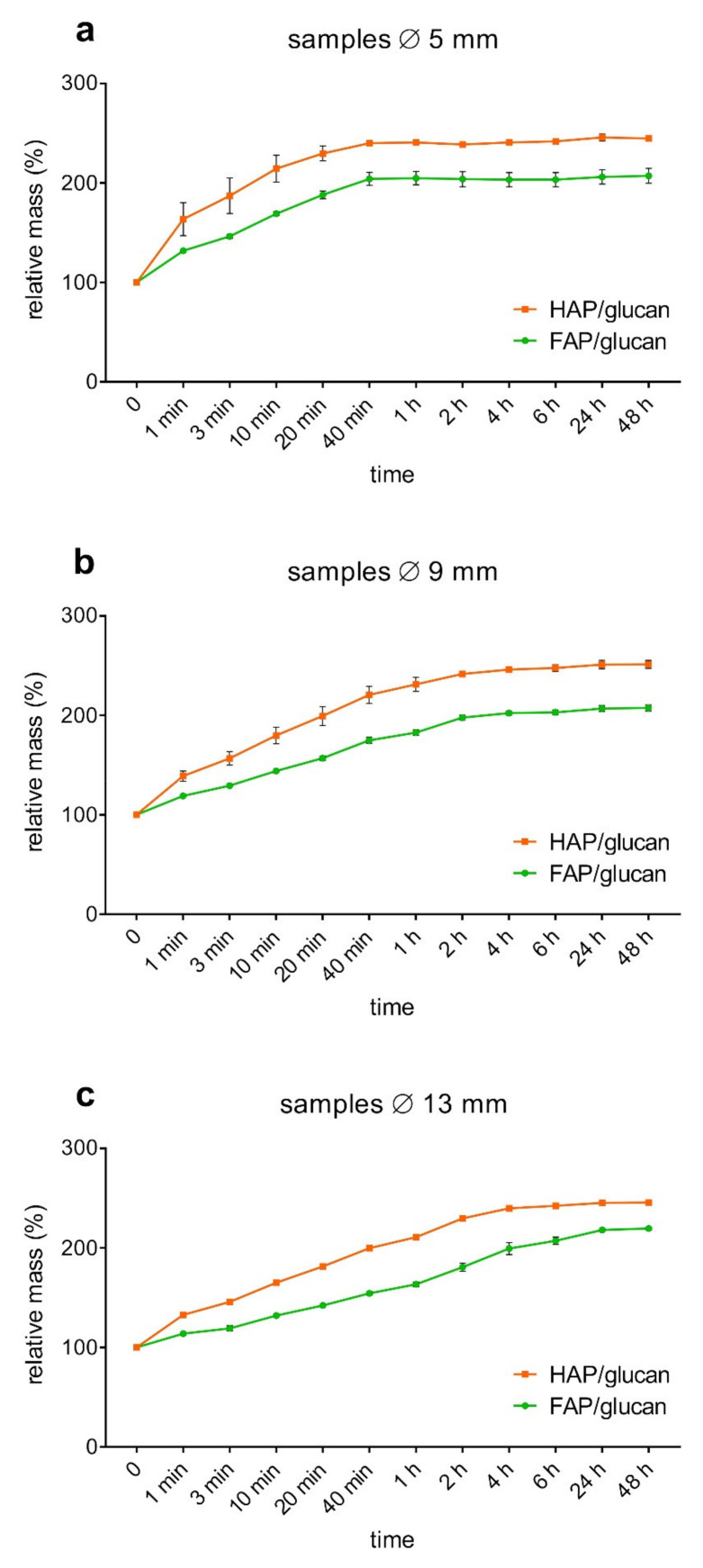
Relative weight change of composites samples soaked in Ringer solution.Panels: (**a**) samples Ø 5 mm, length 9 mm; (**b**) samples Ø 9 mm length 12 mm; (**c**) samples Ø 13 mm length 15 mm.

Soaking capacity of tested materials (of three different dimensions) was determined during 48 h (2880 min.) incubation in Ringer solution. The weight of completely soaked HAP/glucan composite reached 250% of initial dry weight of samples for all sizes. However, it should be noted that rate of soaking was faster for small samples (5 mm diameter) than for medium ones (9 mm diameter) and the slowest for the largest ones (13 mm diameter). For example, weight of soaked samples of ø 5 mm, ø 9 mm and ø 13 mm was approx. 170%, 140% and 130% of dry weight, respectively, after 1 min of soaking (Figure 5). This observation was similar to our earlier observations described elsewhere [43]. For FAP/glucan composite, soaking capacity was in all cases lower than for relevant HAP/glucan ones. After 1 min of soaking, the weight of samples was approx. 130%, 120% and 110% of dry weight, respectively. The weight of completely soaked samples reached 205–220% of initial dry weight of all samples. For both biomaterials, the common tendency was found: the smaller is the sample, the faster is rate of soaking (Figure 5). Also, the dynamics of soaking, at least within the period of the first 40 min of soaking, are similar for HAP/glucan and FAP/glucan composites. Comparing soaking properties of composites based on HAP and FAP, it seems probable that they are result of ceramics porosity. Higher porosity of HAP granules allows for absorption of more water that FAP granules of lower porosity (Figure 3 and Table 1). 

**Figure 6:**
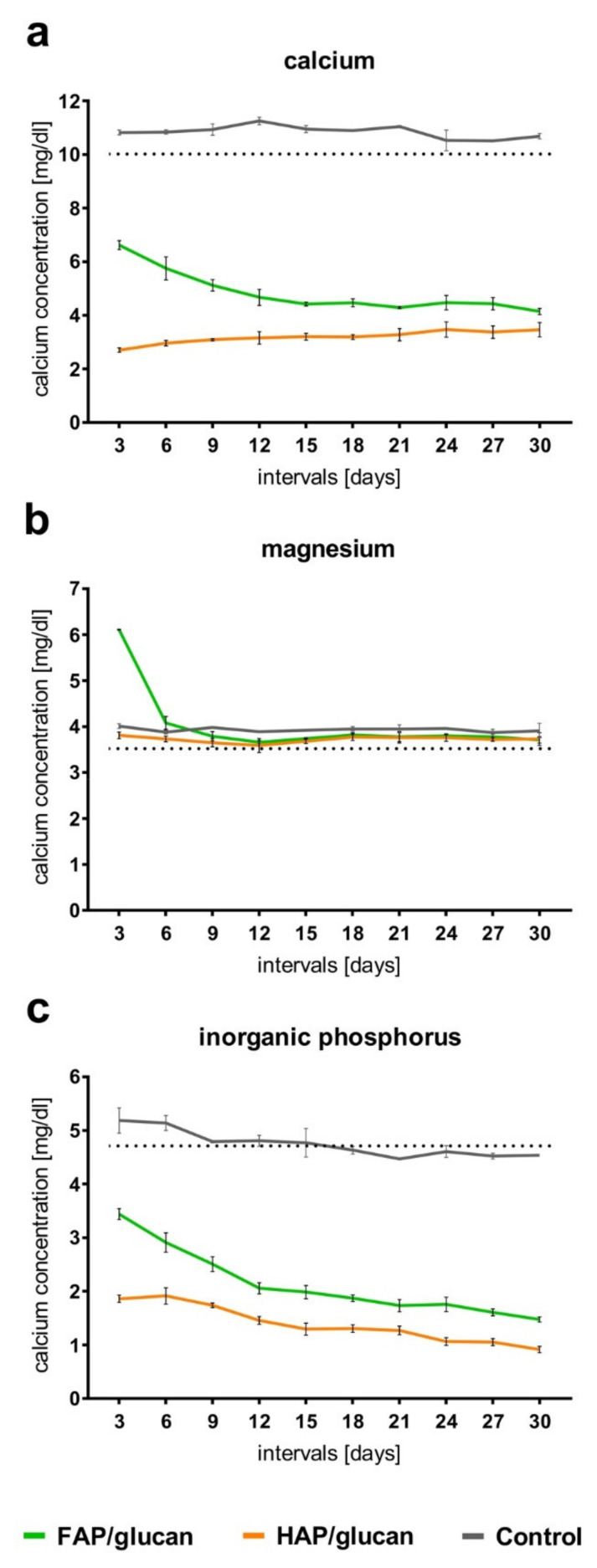
Calcium (**a**), magnesium (**b**), and phosphorus (**c**) ion changes in SBF by investigated materials. Dotted line indicates ion concentration in freshly made fluid.

Incubation of both HAP/glucan and FAP/glucan in SBF for 30 days provided information concerning the uptake of Ca^2+^, Mg^2+^ and (PO_4_)^3−^ ions from the surrounding medium. It was found that calcium and phosphate ions were adsorbed by the surface of both composites during the incubation in SBF. It was noted that in case of both these ions, their adsorption by HAP/glucan composite proceeded more intensely than by FAP/glucan composite (Figure 6). To confirm whether both these ions were built into newly formed apatite deposited onto the composites, EDS analysis of polymeric phase surface was performed (before the incubation in SBF, polymeric phase did not contain calcium and phosphorus). The results (Table 2) showed that both calcium and phosphorus were detected on curdlan polymer layer of the composites incubated 30 days in SBF. The amounts of both these ions detected on the surface of HAP/glucan samples were approximately 2.5 times higher than the amounts on the surface of FAP/glucan samples (Table 2). This observation is in agreement with the results of calcium and phosphate ions uptake from SBF (Figure 6a,c). Overall, the obtained data confirm that apatite is being formed from SBF both on HAP/glucan and FAP/glucan composite. However, this process proceeds faster for HAP/glucan material. This phenomenon is probably related to higher porosity of HAP/glucan composite (as a result of higher porosity of HAP granules than FAP granules) and is in agreement with the data concerning the dynamics of ions uptake from SBF by HAP and FAP ceramic granules reported elsewhere [31].

**Figure 7:**
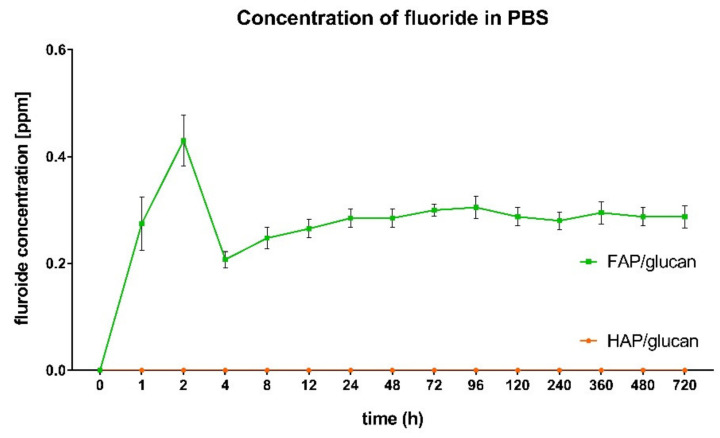
Fluoride release profile from the composite materials to PBS medium with a pH 7.4. The HAP/glucan composite did not release fluoride into the buffer.

Interestingly, some fluorine was observed to be built into the apatite deposited on curdlan layer in FAP/glucan composite (0.29 atomic %; Table 2). This suggests that, in the case of this composite, some type of fluorapatite ceramics is formed during the incubation in SBF. The source of the fluoride ions crucial for this process is FAP/glucan composite—it was shown that some fluoride ions were released from this composite during the incubation in PBS (Figure 7). The burst of F^−^ release was observed during first 2 h of the incubation. Then the level of the released F^−^ ions remained on relatively constant level (0.25–0.3 ppm) during the entire incubation period. The profile of F^−^ ions released from FAP/glucan composite observed in this study was similar to that noted for FAP granules, as reported earlier [31]. The F^−^ ions are therefore likely to be released into other media—tissue liquids (in-vivo) and culture media (in-vitro in cell culture tests). 

Magnesium ions were absorbed from SBF neither by HAP nor by FAP-based composites (Figure 6b, Table 2). It was surprising because calcium and magnesium ions are usually absorbed simultaneously from ions-rich media by calcium phosphate ceramics [43]. More surprising, some magnesium ions were released by FAP/glucan composite to SBF during the first and second medium exchange; afterwards, no further Mg^2+^ release was observed (Figure 6b). Possibly, these magnesium ions were entrapped within FAP granules as post-production impurities and were eluted during initial period of composite incubation in SBF. 

**Figure 8:**
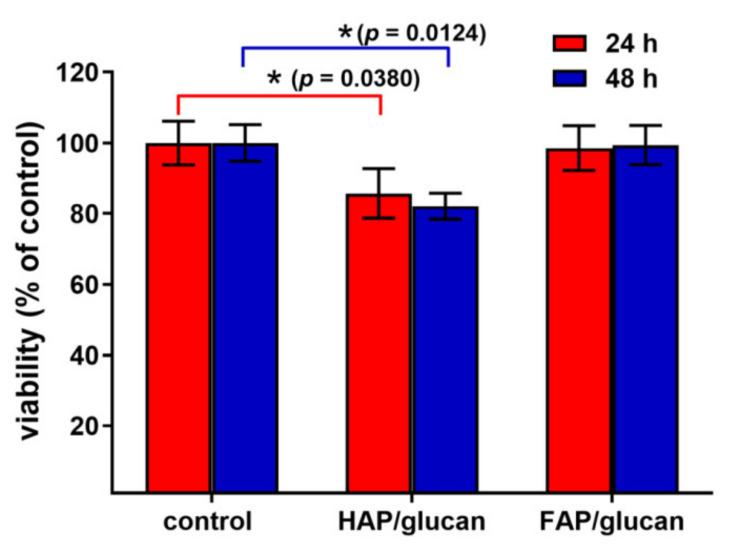
MTT cytotoxicity test performed for the biomaterials using human osteoblasts (hFOB 1.19 cell line) according to ISO 10993-5 standard (* statistically significant results according to unpaird Student’s *t*-test).

**Figure 9:**
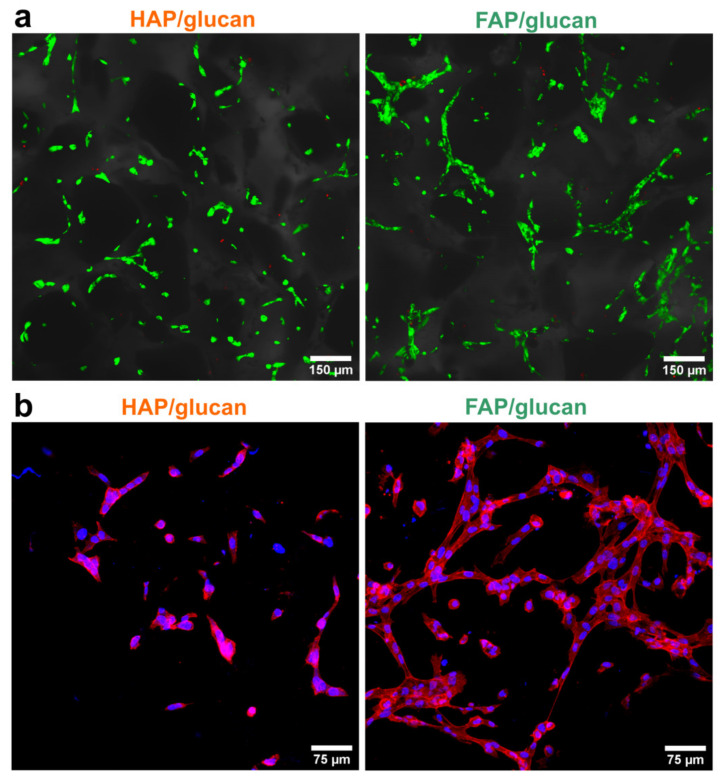
(**a**) Viability of cells in direct contact with the biomaterials visualized using confocal microscope (green fluorescence—viable cells, red fluorescence—dead cells, Nomarski contrast was used to visualize composite microstructure); (**b**) cell growth on the surface of the biomaterials visualized after fluorescent staining of cytoskeleton (red fluorescence) and nuclei (red fluorescence).

Cytotoxicity test performed according to ISO 10993-5 revealed that FAP/glucan composite is non-toxic (cell viability near 100%), whereas HAP/glucan biomaterial significantly reduced cell viability to 85% (24-h exposure) and 82% (48-h exposure) compared to the control cells (Figure 8). Nevertheless, according to ISO 10993-5 standard HAP/glucan materials should be still considered as non-toxic since cell viability was higher than 70%. Live/dead fluorescent staining confirmed the results obtained with MTT assay. Cells were viable and thus showed mainly green fluorescence. There were only single dead cells showing red fluorescence of nuclei (Figure 9a). However, noticeably fewer cells were observed on the surface of HAP/glucan compared to FAP/glucan composite. Visualization of cell growth after 48-h culture on the composite structure (cytoskeleton staining) also revealed meaningfully smaller number of osteoblasts with less extensive cytoskeleton structure on the HAP/glucan material compared to the composite enriched with fluoride ions (Figure 9b). All these observations suggested that FAP/glucan biomaterial was more favorable to osteoblast adhesion and growth, and thus showed better osteoconductivity.

**Figure 10:**
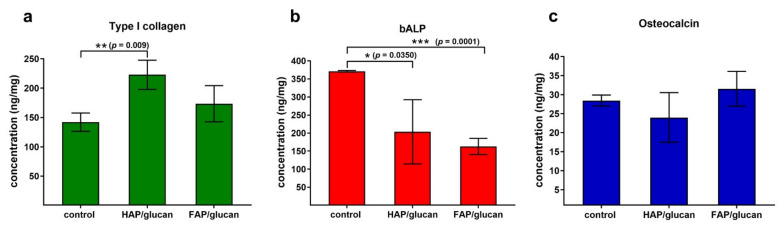
Osteogenic differentiation markers assessed by ELISAs for osteoblasts cultured on the biomaterials and polystyrene well as a control: (**a**) type I collagen, (**b**) bone alkaline phosphatase, (**c**) osteocalcin. The level of osteogenic markers was normalized to 1 mg of cellular proteins (assessed by BCA assay). (*, **, *** statistically significant results according to unpaird Student’s *t*-test).

Since meaningfully fewer cells were attached to HAP/glucan material compared to FAP/glucan composite, osteogenic differentiation potential of the cells grown on the samples was estimated after normalization of the level of bone formation markers to 1 mg of cellular proteins. This approach allowed to exclude an error related to different cell number on the samples. Analysis of osteogenic differentiation process showed that osteoblasts grown on the HAP/glucan produced significantly greater amounts of Col I compared to the control cells (Figure 10). Although cells on FAP/glucan synthesized smaller amount of this protein compared to cells on HAP/glucan, there were no statistically significant results between mentioned samples. Osteoblasts cultured on both composites showed significantly lower level of bALP compared to the control cells. In the case of osteocalcin, no statistically significant differences were observed between the samples. The greatest difference between the samples was observed for bLAP marker. It should be noted that bALP is an early marker of osteogenic differentiation. It is detectable during the first phase (proliferation) and its highest activity is observed during the second phase (extracellular matrix synthesis) of osteogenic differentiation [44]. Based on obtained results, it may be assumed that control cells were still in the late second phase, whereas cells on the composites started the third phase (extracellular matrix mineralization) of the differentiation, which is characterized by decreased bALP activity. It is known that OC release is correlated with calcification of extracellular matrix due to its ability to bind hydroxyapatite. Thus, higher OC production (but without statistical significance) by cells on the FAP/glucan also indicates that osteoblasts most likely started the mineralization phase.

## 3. Materials and Methods

### 3.1. Materials 

FAP and HAP ceramic granules were produced according to patent procedures no. PL 235803 and no. EP 2229961, respectively [30,45]. The method of producing FAP granules and its physicochemical and biological properties were described in [31]. Briefly, FAP and HAP were prepared by the sol-gel method and calcined in a furnace for 2 h at a temperature of 800 °C. Sintered ceramics were ground in a mortar and separated on a sieve to obtain granules of 0.2–0.3 mm.

β-1,3-glucan (curdlan) from *Alcaligenes faecalis* (DP 450) was supplied by Wako PureChemical Industries (Osaka, Japan).

### 3.2. Preparation of Samples (FAP/glucan and HAP/glucan Composites)

FAP and HAP granules were used to prepare two types of ceramic/polymer composites, these were as follows:

- FAP/glucan
- HAP/glucan

FAP/glucan composite samples were synthesized according to the procedure described in Polish Patent no. 236369 [32]. Briefly, FAP granules were combined with aqueous suspension of β-1,3-glucan (dry weight proportion: 83wt.% granules and 17wt.% β-1,3-glucan). The resulting mixture was baked (95 °C, 15 min.) in moulds, cut into samples, dried (72 h, 25 °C) and finally sterilized in plastic/paper peel pouch by exposure to ethylene oxide for 1 h at temp. 55 °C, followed by 20 h of aeration. 

HAP/glucan composite was produced according to European Patent no. EP 2421570 and served as a reference for FAP/glucan [46]. 

The shape and size of the samples were adapted to each experiment (e.g., apparatus limitations, guidelines according to ISO standards) and were summarized in Table 3.

### 3.3. Characterization of Biomaterials

#### 3.3.1. SEM Imaging

Composites’ cross-sections were visualized using scanning electron microscope model JCM-6000Plus (JEOL, Tokyo, Japan). The samples were sputtered with 8 nm thick gold layer with the use of sputter coater model DSR1 (Vac Coat, London, UK). The SEM imaging was performed at an accelerating voltage of 5 kV and in a high vacuum environment.

#### 3.3.2. FTIR Analysis

The FTIR-ATR spectra (diamond crystal) were obtained using an IR spectrometer model Vertex 70 (Bruker Corporation, Billerica, MA, USA), 64 scans, 4 cm^−1^ resolution. Data were analyzed by the Opus 7.0 software (Bruker Corporation, Billerica, MA, USA).

#### 3.3.3. Porosity Determination

The Autopore IV 9500 mercury porosimeter (Micrometrics Inc., Norcross, GA, USA) was used to measure the most frequent porosimetric parameters according to the ISO 15901-1:2005 standard [47]. Before the experiment, the samples (*n* = 3) were dried at 37 °C. Mercury intrusion was performed at pressures up to 228 MPa. The range of the examined pores was 5 nm to 360 µm.

#### 3.3.4. Mechanical Property Evaluation by Compression Test

The behavior of the composites during compression was assessed on dry and wet samples (after a 24-h soaking in deionized water). The Autograph AG-X plus testing machine (Shimadzu, Kyoto, Japan) was used to conduct compression testing with pre-load value of 1 N with crosshead moving speed 10 mm/min, followed by basic load rate of 0.5 mm/min. Load rate was lower than standardized for most of materials because of viscoelasticity nature of tested composites. The mechanical compression was carried out until 30% of strain was reached and the obtained data allowed the stress-strain characteristics.

#### 3.3.5. Liquid Absorption Behavior of Composites

In order to determine water uptake behaviour, the composite samples of various sizes (Table 3) were immersed in Ringer’s solution. Two types of composites of three different sizes were tested, which gives a total of 6 independent groups (*n* = 3). Three samples from each group were placed individually in wells of sterile culture plate and soaked in 10 mL of Ringer’s solution (990 mL of ultrapure water + 8.6 g NaCl + 0.3 g KCl + 0.48 g CaCl_2_). Incubation was carried out for 48 h at 37 °C to simulate the temperature of human body. All samples were weighed on an analytical balance with accuracy 0.00001 g (XS205, Mettler-Toledo, Switzerland) before and during incubation in solution at defined time points (after 1, 3, 10, 20, 40 min and 1, 2, 4, 6, 24, 48 h). Excess of the fluid was removed before weight measurement using Whatman article.

#### 3.3.6. Bioactivity Assessment

The apatite-forming ability of the biomaterials was estimated in accordance with ISO 23317 procedure by soaking of the samples in a simulated body fluid (SBF). The SBF (pH 7.25) was prepared according to method proposed by Kokubo et al. 1990 [48]. Afterwards, the fluid was sterilized by mechanical filtration, using the Stericup filter (500 mL, 0.22 µm; Millipore Corporation) under vacuum.

Disc-shaped FAP/glucan samples (*n* = 4) and HAP/glucan samples (*n* = 4) were placed separately in 6-well plate and incubated in 10 mL of SBF at 37 °C for 30 days. The SBF solution was exchanged every 3 days to ensure sufficient ion concentrations for mineral growth. In SBF collected every 3 days, the level of Ca^2+^ and Mg^2+^ ions as well as inorganic phosphorus (as a measure of (PO_4_)^3−^ ions) was evaluated using commercial kits: “Calcium CPC”, “Magnesium” and “Phosphorus” acquired from BioMaxima Inc. (Lublin, Poland). Composition of composite surface after 30 days incubation in SBF was estimated by means of EDS method. The regions of interest (polymer phase of composites) for EDS analysis were determined using high-resolution SEM images. The chemical composition was presented in atomic percent (at. %).

#### 3.3.7. Fluoride Ion Release Test

FAP/glucan and HAP/glucan composite samples were tested for fluoride ion release level. The size of the samples was selected to contain 1 g of granules. Four samples for each group were prepared (*n* = 4). 

All samples were placed in Falcon 50 mL tubes, immersed in 10 mL of sterile PBS buffer, and incubated at 37 °C for 30 days. At defined time points (after 0; 1; 2; 4; 8; 12; 24; 48; 72; 96; 120; 240; 360; 480; 720 h), 2 mL of PBS was taken out from each tube in order to determine concentration of fluoride ions (followed by supplementation with 2 mL fresh PBS solution). Then, 2 mL of Total Ionic Strength Adjustment Buffer (TISAB) was added to each sample to be determined for fluoride concentration. A fluoride-selective electrode model Orion 9609-BNWP (Thermo Scientific, Waltham, MA, USA) in combination with a multi-meter CPI-505 (Elmetron, Zabrze, Poland) was applied to measure concentration of F^−^. The data shown has been corrected for the amount of fluoride removed from the incubation tubes to measure its concentration.

### 3.4. In-Vitro Cell Culture Experiments

Cell culture experiments were performed using human normal fetal osteoblast cell line (hFOB 1.19, ATCC). The cells were cultured in a 1:1 mixture of DMEM/Ham’s F12 medium (Sigma-Aldrich, Germany) with the following supplementation: 10% fetal bovine serum (FBS, Pan-Biotech), 100 U/mL penicillin, 0.1 mg/mL streptomycin, and 0.3 mg/mL G418 (all antibiotics were purchased from Sigma-Aldrich Chemicals). The culture conditions were as follow: 34 °C, 5% CO_2_, 95% air, humidified atmosphere.

#### 3.4.1. Quantitative Cytotoxicity Assessment (MTT)

The cytotoxicity test was conducted according to ISO 10993-5:2009 standard [49]. Briefly, 2 × 10^4^ of hFOB 1.19 cells were seeded into the wells of 96-multiwell plates. After 24-h incubation, the culture medium was discarded and extracts of the biomaterials were added. The extracts were prepared according to ISO 10993-12:2012 standard by immersion of biomaterials (for 24 h at 37 °C) in the complete culture medium, keeping the ratio 1 mL of the medium per 100 mg sample [50]. Cells maintained in the culture medium that was incubated in the same condition but without biomaterial were considered as negative control, exhibiting 100% viability. Osteoblasts were exposed to the extracts for 24 h and 48 h and then cell viability was evaluated by MTT assay (Sigma-Aldrich Chemical) as it was described in detail previously [51]. The cytotoxicity was assessed in 4 independent experiments. Statistically significant results were considered at *p* < 0.05 according to an unpaired *t*-test (GraphPad Prism 8.0.0 Software, San Diego, CA, USA). 

#### 3.4.2. Direct-Contact Test for Qualitative Cytotoxicity Assessment (Live-Dead Staining)

Biomaterial samples were presoaked in the complete culture medium for 1 h. Then, 1 × 10^5^ cells were seeded directly on top surface of the composites. After 48-h culture of the cells on the composites, their viability was determined by Live/Dead Double Staining Kit (Sigma-Aldrich, Germany) and confocal laser scanning microscope observation model Fluoview equipped with FV1000 (Olympus, Tokyo, Japan). The staining procedure was performed according to manufacturer instruction using calcein-AM and propidium iodide fluorescent dyes. 

#### 3.4.3. Cell Growth Evaluation (Cytoskeleton Imaging—DAPI, Phalloidin)

Cell growth on the composites was determined by seeding 1 × 10^5^ osteoblasts on the surface of the biomaterial and their observation using CLSM after fluorescent staining of cytoskeleton and nuclei. The osteoblasts were cultured on the composites for 48 h at 37 °C and then the cells were fixed and stained using AlexaFluor635-conjugated phallotoxin (Invitrogen, Grand Island, NY, USA) and DAPI (Sigma-Aldrich, Germany) as it was described previously [52].

#### 3.4.4. Osteogenic Differentiation Evaluation (ELISAs)

The osteoblasts were seeded directly on the biomaterials as it was described in Section 3.4.2. Cells cultured on the polystyrene wells in 48-multiwell plate served as a control. After 24-h culture, the medium was discarded and osteogenic differentiation of the cells was stimulated by addition of the medium enriched with: 10 mM β-glycerophosphate, 0.05 mg/mL ascorbic acid, and 10^−8^ M dexamethasone (all supplements were purchased from Sigma-Aldrich, Germany). Half of the osteogenic medium was replaced with fresh portion every 3–4 days. After stimulation of cells for 13 days, cell lysates were prepared by 2 freeze-thaw cycles and sonication as it was previously described [44]. The level of type I collagen (Col I), bone alkaline phosphatase (bALP) and osteocalcin (OC) was measured using human-specific ELISA kits purchased from EIAab (Col I, OC) and FineTest (bALP). The level of each marker of osteogenic differentiation was normalized to the 1 mg of the cellular proteins. For this purpose, total protein content was assessed for each sample by colorimetric method using BCA Protein Assay Kit (Thermo Fisher Scientific, Rockford, IL, USA). Evaluation of the osteogenic differentiation was performed using 3 independent samples of the biomaterials. Statistically significant results were considered at *p* < 0.05 according to an unpaired *t*-test (GraphPad Prism 8.0.0 Software, San Diego, CA, USA). 

## 4. Conclusions

Our research presented the properties of the fluorapatite/glucan composite material with particular emphasis on biological potential in terms of application in orthopedics and bone tissue engineering. This work also discussed the differences between FAP/glucan and HAP/glucan composites. Type of granules used in the production of biomaterials determined the porosity, pore size distribution, mechanical properties, liquid absorption, and apatite forming ability. As expected, fluoride-containing biomaterial exhibited lower porosity, lower total pore area, and higher density, which resulted in lower water absorption compared to the reference material. Presumably, due to the greater chemical stability of FAP than HAP, the FAP/glucan composite showed lower apatite forming ability also confirmed by a smaller loss of calcium and phosphate ions from the SBF fluid. Fluorapatite granules contained in the FAP/glucan composite were found to gradually release fluoride ions into the surrounding fluid, at a level not exceeding the toxic value for osteoblasts cell line which is estimated <1 ppm. The use of FAP instead of HAP in the ceramic-polymer composite has brought biological benefits demonstrated during in vitro cell culture experiments. Our study revealed that the properties of FAP/glucan positively influenced osteoblast growth, adhesion and increased osteocalcin synthesis as compared to the HAP/glucan composite. Summarizing the above results, the fluorapatite-glucan composite has great potential for use in regenerative medicine in the field of traumatic orthopedics. Due to the ability to release fluoride ions in optimal amounts, the biomaterial has a positive effect on the adhesion of osteogenic cells and their multiplication, which may result in bone reconstruction in the event of its implantation at the site of injury. Taking into account the positive properties of the new bone substitute material based on fluoroapatite, it can be assumed that it will be a good implantable material contributing to the acceleration of the bone regeneration process at the site of the defect. However, to confirm this, it is necessary to conduct in-vivo experiments with laboratory animals, and then with the participation of patients in bone trauma surgery clinics.

## 5. Patents

The scaffold and the method for the production of the scaffolds were claimed in the Polish Patent. Borkowski, L.; Belcarz, A.; Przekora, A.; Ginalska, G. Method of Obtaining Bone Scaffold based on Fluoroapatite Ceramics and Polymer and Bone Scaffold. Polish Patent no. 236369, 29.01.2021 [32].

## Figures and Tables

**Table 1:** Selected parameters of porosity of FAP/glucan and HAP/glucan composites.

Parameter	Sample		Statistically Significant Difference *
	FAP/Glucan Mean (±SD)	HAP/Glucan Mean (±SD)	
Total intrusion volume [cm^3^/g]	0.47 (±0.004)	0.88 (±0.014)	yes, *p* ≤ 0.0001
Total pore area [m^2^/g]	5.16 (±0.21)	20.78 (±0.3)	yes, *p* ≤ 0.0001
Bulk density [g/cm^3^]	1.14 (±0.006)	0.75 (±0.01)	yes, *p* ≤ 0.0001
Apparent (skeletal) density [g/cm^3^]	2.43 (±0.035)	2.20 (±0.05)	yes, *p* ≤ 0.01
Porosity [%]	53.19 (±0.58)	65.93 (±0.32)	yes, *p* ≤ 0.0001
Median pore diameter (Volume based) [µm]	1.41 (±0.11)	0.13 (±0)	yes, *p* ≤ 0.0001
Median pore diameter (Area based) [µm]	0.21 (±0.006)	0.1 (±0)	yes, *p* ≤ 0.0001
Average pore diameter [µm]	0.36 (±0.015)	0.17 (±0.006)	yes, *p* ≤ 0.0001

* statistically significant differences between FAP/glucan and HAP/glucan composites according to unpaired Student’s *t*-test.

**Table 2:** Mean element content (expressed as atomic percent) on polymeric surface of materials according to EDS analysis.

	Calcium		Phosphorus		Carbon		Oxygen		Fluorine	
	Mean	SD	Mean	SD	Mean	SD	Mean	SD	Mean	SD
HAP/glucan	4.27	2.13	2.59	1.01	49.12	4.87	44.03	2.58	0	0
FAP/glucan	1.74	1.53	0.98	0.58	53.84	3.21	43.14	1.53	0.29	0.27
statistically significant difference *	yes, *p* ≤ 0.0001		yes, *p* ≤ 0.0001		yes, *p* ≤ 0.01		no, *p* > 0.05		yes, *p* ≤ 0.0001	

* statistically significant differences in the element content between FAP/glucan and HAP/glucan composites according to Mann-Whitney test.

**Table 3:** Shape and size of composites samples used in the experiments.

Experiment	Sample		
	Shape	Diameter (mm) (± 0.5 mm)	Height (mm) (± 0.5 mm)
SEM imaging, Live-Dead staining, Cytoskeleton Imaging, ELISA tests	flat disc	8	2
Bioactivity assessment	flat disc	10	2
Absorbability testing	cylindrical	5	9
		9	12
		13	15
Mechanical testing	cylindrical	8	8
Porosimetry testing, Fluoride ion release test	cylindrical	11	11

## Data Availability

All important data is included in the manuscript.
